# Use of the HoloLens2 Mixed Reality Headset for Protecting Health Care Workers During the COVID-19 Pandemic: Prospective, Observational Evaluation

**DOI:** 10.2196/21486

**Published:** 2020-08-14

**Authors:** Guy Martin, Louis Koizia, Angad Kooner, John Cafferkey, Clare Ross, Sanjay Purkayastha, Arun Sivananthan, Anisha Tanna, Philip Pratt, James Kinross, PanSurg Collaborative

**Affiliations:** 1 Department of Surgery and Cancer Imperial College London London United Kingdom; 2 Division of Surgery Imperial College Healthcare NHS Trust London United Kingdom; 3 Cutrale Perioperative and Ageing Group Imperial College London London United Kingdom; 4 Division of Medicine, Imperial College Healthcare NHS Trust London United Kingdom; 5 West London Renal and Transplant Centre, Imperial College Healthcare NHS Trust London United Kingdom; 6 The Helix Centre Imperial College London London United Kingdom; 7 See Authors' Contributions section

**Keywords:** COVID-19, mixed reality, telemedicine, protection, acceptability, feasibility, impact, headset, virtual reality, augmented reality, pilot

## Abstract

**Background:**

The coronavirus disease (COVID-19) pandemic has led to rapid acceleration in the deployment of new digital technologies to improve both accessibility to and quality of care, and to protect staff. Mixed-reality (MR) technology is the latest iteration of telemedicine innovation; it is a logical next step in the move toward the provision of digitally supported clinical care and medical education. This technology has the potential to revolutionize care both during and after the COVID-19 pandemic.

**Objective:**

This pilot project sought to deploy the HoloLens2 MR device to support the delivery of remote care in COVID-19 hospital environments.

**Methods:**

A prospective, observational, nested cohort evaluation of the HoloLens2 was undertaken across three distinct clinical clusters in a teaching hospital in the United Kingdom. Data pertaining to staff exposure to high-risk COVID-19 environments and personal protective equipment (PPE) use by clinical staff (N=28) were collected, and assessments of acceptability and feasibility were conducted.

**Results:**

The deployment of the HoloLens2 led to a 51.5% reduction in time exposed to harm for staff looking after COVID-19 patients (3.32 vs 1.63 hours/day/staff member; *P*=.002), and an 83.1% reduction in the amount of PPE used (178 vs 30 items/round/day; *P*=.02). This represents 222.98 hours of reduced staff exposure to COVID-19, and 3100 fewer PPE items used each week across the three clusters evaluated. The majority of staff using the device agreed it was easy to set up and comfortable to wear, improved the quality of care and decision making, and led to better teamwork and communication. In total, 89.3% (25/28) of users felt that their clinical team was safer when using the HoloLens2.

**Conclusions:**

New technologies have a role in minimizing exposure to nosocomial infection, optimizing the use of PPE, and enhancing aspects of care. Deploying such technologies at pace requires context-specific information security, infection control, user experience, and workflow integration to be addressed at the outset and led by clinical end-users. The deployment of new telemedicine technology must be supported with objective evidence for its safety and effectiveness to ensure maximum impact.

## Introduction

The coronavirus disease (COVID-19) pandemic has overwhelmed even the most developed and well-resourced health systems [1]. Difficult decisions regarding the rationing of personal protective equipment (PPE) for health care workers and even access to care for patients have had to be made [2]. Protecting the health and safety of care workers is a key priority to maintain the quality of care delivered to individual patients and the ability of health systems to deliver care at scale [3]. In Italy, up to 20% of health care workers have become infected with the virus [4], and in the United Kingdom, over 15% of positive tests have been in critical health care workers [5] and over 100 have died [6]—a picture that has been seen globally. A key aspect of this has been severe disruption and shortages in the global supply of PPE due to excess demands and misuse [7]. As such, novel methods that optimize PPE use and protect both health care workers and patients from COVID-19 transmission are urgently needed [8].

Digital innovation has been identified as key to tackling the challenge to staff safety that COVID-19 confers [9]. The pandemic has rapidly accelerated the deployment of new technologies such as telemedicine services [10]. Telemedicine provides a means to deliver care efficiently and leverage access to multiple remote specialists while simultaneously protecting staff and patients from exposure to the virus [11,12]. Mixed-reality (MR) technology offers an immersive experience in which real and virtual elements of an environment dynamically coexist; it is the most recent iteration and extension of telemedicine innovation with the potential to revolutionize clinical care and education through the provision of enhanced functionality and novel content.

The HoloLens2 is an untethered wearable holographic computer that allows bidirectional communication with multiple remote users via video, voice, and MR composites. The technology has been used previously in a variety of clinical and educational scenarios, including perioperative planning, surgical training, anatomical teaching, and 3D telemedicine support [13-17]. The technology offers the potential to increase user immersion and engagement, supplement situational awareness and access to knowledge in real time, and improve performance. It allows users to interact and manipulate spatially registered 3D holographic content within a real environment, and to remotely link with multiple devices and users to allow simultaneous collaborative interaction and working within the visualized environment. MR technology is in its infancy but is the logical next step in the move toward the provision of digitally supported clinical care and education.

The HoloLens2 device has not previously been deployed for the delivery of ward-based secondary care in high-risk environments. This technology-led pilot therefore deployed and evaluated the HoloLens2 for the delivery of remote care across a range of inpatient settings in a UK teaching hospital during the COVID-19 pandemic response. The aim was to assess the practicalities and impact of deploying MR telemedicine technologies for improving staff safety during the pandemic.

## Methods

### Conduct

The objective of this project was to assess the practicalities and clinical impact of introducing a remote distributed care model supported by the HoloLens2. All technologies sit within a multidomain system that includes its users and environment; it cannot be developed, deployed, or evaluated in isolation [18]. Workflow considerations and human factors must therefore also be considered alongside technical decisions when understanding the practicalities and impact of implementing a digital technology and models of care at pace.

A prospective, observational, nested cohort evaluation of the HoloLens2 as a technology-led quality improvement (QI) project was performed. The device was deployed and evaluated across three distinct clinical clusters: a COVID-19 general medicine ward, a specialist COVID-19 unit providing continuous positive airway pressure support, and finally a specialist unit providing care to COVID-19 patients with renal disease. High-level aggregate outcome data pertaining to staff exposure to high-risk COVID-19 environments and PPE use were collected. Assessments of feasibility and acceptability were undertaken via user experience questionnaires with Likert and free-text responses. Local institutional registration and approval was obtained, and data governance and infection prevention and control procedures were agreed upon prior to the commencement of the project. No additional ethical approval was required as the project was conducted as a technology-led QI project under the supervision of the institutional QI team. Explanatory information was provided to all participants. All data were arranged, structured, and analyzed in Microsoft Excel (V15.22, Microsoft Corporation) and IBM SPSS (V26, IBM Corporation). With regards to statistical analysis, standard descriptive statistics were employed, and two-tailed Student’s *t* tests were used to compare differences, with significance set at *P*<.05.

### Workflow

Prior to the deployment of the HoloLens2, standard clinical practice in all three areas was to provide clinical care through face-to-face ward rounds comprising 3-8 clinical staff members. This required all to don appropriate PPE and provide care in high-risk environments. For this project clinical teams were provided with standardized training on the basic functions of the HoloLens2 device. Teams were not given specific instructions on how to alter their clinical practice or models of care and were free to use the technology in an optimal way for their local clinical context to support the transition to a remote distributed care model.

**Figure 1:**
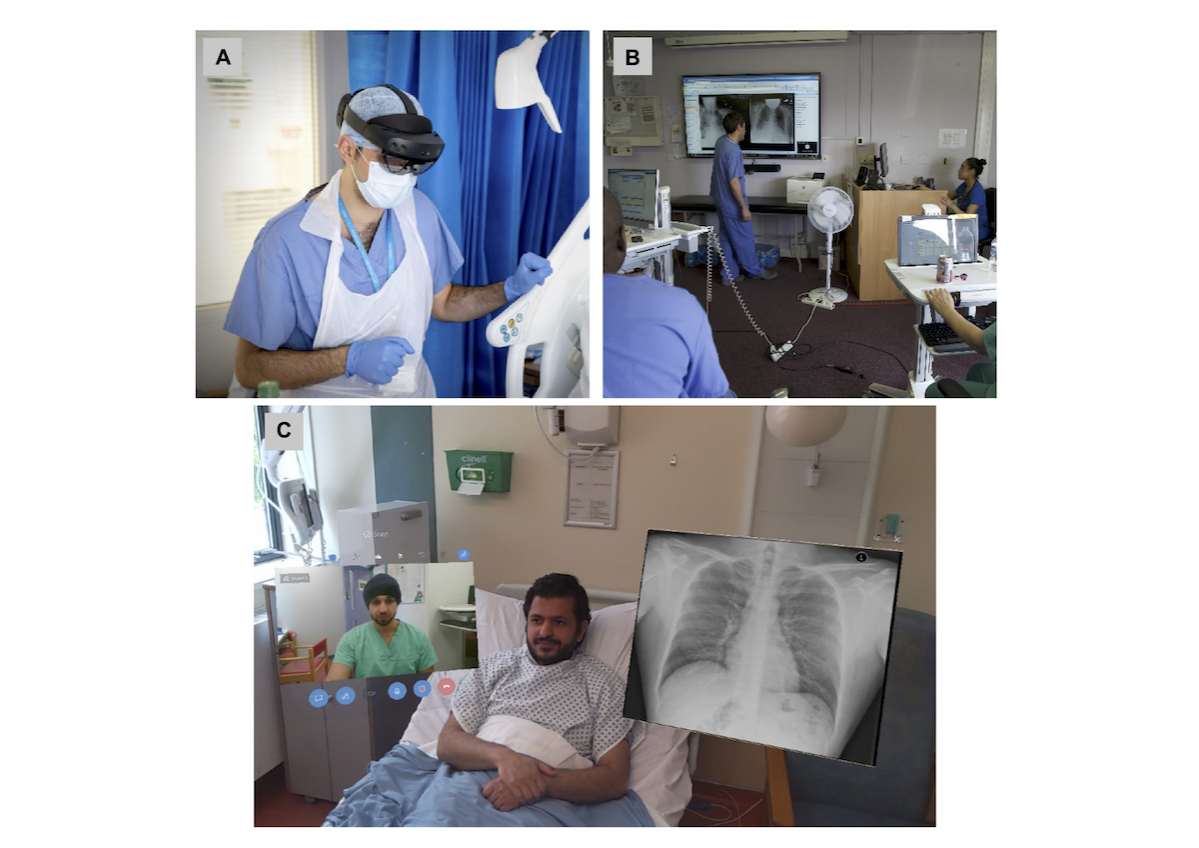
Images demonstrating the use and functionality of the HoloLens2 device. (A) View of end-user in personal protective equipment wearing the device. (B) View of remote clinical team engaging in clinical round from a safe location. (C) First-person view through the HoloLens2 showing the remote clinical team and relevant imaging placed in the user’s field of view as mixed-reality composites (image generated with study group to ensure the protection of patient privacy and data).

In general, following deployment a single senior member of staff would enter the COVID-19 environment to undertake rounds, with the remainder of the team joining virtually from a COVID-19–protected, nonclinical remote location. All members of the team then played an active role in clinical assessment and decision making through the bidirectional audio-visual functionality of the device, and specifically through the first-person bedside view provided to the remote team members. In addition, relevant imaging and electronic health record (EHR) data were placed directly into the field of vision of the device user, with the aim of improving situational awareness, informing better clinical decision making and further reducing the risk of viral transmission by minimizing the need to interact physically with equipment and technology in high-risk areas. In parallel, members of the remote team would document encounters in the EHR, and undertake electronic ordering and prescribing in real time. Device use and functionality is illustrated in Figure 1.

### HoloLens2

The HoloLens2 is produced and marketed by Microsoft Corporation (Redmond, WA, USA) and is an untethered mixed-reality headset that combines several types of sensors, infrared time-of-flight depth measurement, high-definition cameras, accelerometers, and microphones. It provides a true heads-up display functionality with the ability to place interactive 2D and 3D objects, such as medical imaging or EHR data, within a user’s visual field. Simultaneously, it provides live bidirectional communication via video and voice with multiple remote users through the Remote Assist application to enable hands-free multidisciplinary telemedicine at the bedside. Microsoft Dynamic 365 Remote Assist utilizes the architecture of Microsoft Teams—a unified communication and collaboration platform that combines chat, video meetings, file storage, and application integration—which was deployed across the National Health Service (NHS) in England to help providers respond to the COVID-19 pandemic [19]. The deployment of existing well-used and flexible platforms is likely to be beneficial as their technical and security considerations are already understood.

### Information Security and Governance

Ensuring appropriate information security and governance is vital when utilizing new digital technologies, especially when sensitive patient data may be shared electronically. In the United Kingdom, NHSX and other relevant bodies recently updated their guidelines on the use of digital technology and sharing of data during the pandemic, recognizing that a pragmatic, risk-based approach needs to be taken and that an effective pandemic response will require new workflows [20]. For the deployment of the HoloLens2 and Remote Assist, we connected devices to the secure hospital Wi-Fi network; devices were specifically white listed by media access control address (MAC address) and secured by WPA2 preshared key authentication. We utilized mobile device management to automate deployment, provisioning, policy management, application delivery, and updates across all devices. User accounts were protected with strong passwords and multifactor authentication. Institutional approval for data protection, confidentiality, and information sharing was obtained.

### Infection Prevention and Control

The aim of this project was to protect staff from infection; therefore, ensuring a standardized process for wearing the device with PPE and for device decontamination was vital. Specific PPE requirements varied according to each clinical setting, local risk assessment, and clinical tasks being undertaken. However, all members entering a high-risk environment are required to wear 4-5 distinct items of PPE, including gown or apron, gloves, hat, mask or respirator, and eye protection. A pragmatic, risk-based approach to wearing and decontaminating the HoloLens2 devices was developed, with a standard method for using the device with different PPE that included the use of surgical caps to protect the device, as well as customized full-face visors for higher risk areas that had a strip cut out to ensure that the sensors and cameras on the headset were not obstructed, while ensuring adequate protection for staff. The cleaning process was aligned with that used for the decontamination of reusable items of PPE such as full-face plastic visors. The development of safe ways to wear and decontaminate the devices was conducted in partnership with our organizational Infection Prevention and Control team prior to commencing the project. An example of donning/doffing and decontamination procedures has been provided in Multimedia Appendix 1.

## Results

In total, 52 inpatient beds were included across the three clinical clusters, with an aggregate of 51 days of clinical care evaluated during the project. A total of 28 members of clinical staff (n=16, 57.1% male; n=18, 64.3% <35 years of age) completed assessments of acceptability and feasibility, 20 (71.4%) of whom had no experience using the HoloLens2 prior to the project; 23 (82.1%) participants had never used video calling or telemedicine software before the COVID-19 pandemic.

**Figure 2:**
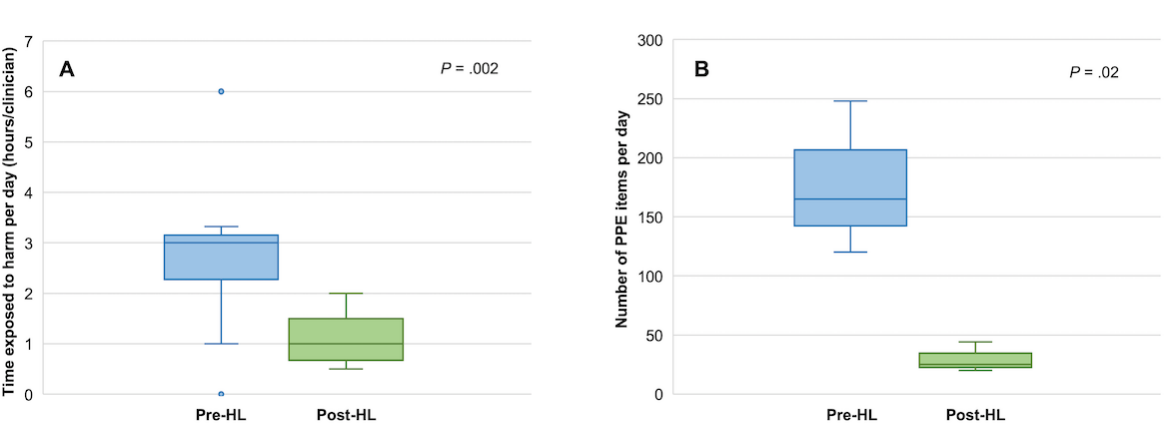
Aggregate data on staff exposure to risk and personal protective equipment (PPE) use across three clinical areas before and after HoloLens2 (HL) deployment. (A) Reduction in time (hours/day/staff member) exposed to high-risk coronavirus disease (COVID-19) environments (3.32 vs 1.63 hours, *t*=3.21, *P*=.002). (B) Reduction in the mean number of PPE items used (178 vs 30, *t*=3.88, *P*=.02).

Deployment of the HoloLens2 led to a significant reduction in the mean aggregate duration (hours/day/staff member) that clinical staff were exposed to high-risk COVID-19 environments while delivering clinical care (3.32 hrs vs 1.63 hrs, *t*=3.21, *P*=.002) as shown in Figure 2A. The total reduction in exposure ranged from 7.15 hrs to 15.45 hrs per day collectively for each team. This represents 222.98 hrs per week of reduced staff exposure to COVID-19 infection across the three clinical areas evaluated, equivalent to a 51.5% reduction. The overall reduction in exposure to risk was achieved by reducing the number of staff on each round by 2-6 people and by greatly reducing the duration of each round. In addition, by allowing the completion of administrative tasks that were previously undertaken following completion of the round in parallel, overall efficiency was increased and postround workload was reduced:

Time was saved by not waiting for other people to change PPE between patients and having to repeat the plan on coming out for bays/side rooms. By the time the ward rounds were complete, the documentation and most of the scans/reviews had been requested.

Renal medicine consultant

Deployment of the HoloLens2 also led to a significant reduction in the mean number of PPE items used (items/round/day) while delivering clinical care in high-risk COVID-19 environments (178 vs 30, *t*=3.88, *P*=.02), as shown in Figure 2B. The total reduction in PPE items used ranged from 100 to 204 per day for each team. This represents a total saving of approximately 3100 items of PPE per week across the three clinical areas evaluated, which is equivalent to an 83.1% reduction. Importantly, 89.3% (25/28) of clinical staff felt that their team was safer when using the HoloLens2 to look after COVID-19 positive patients. No member of staff reported any safety concerns while using the device, and all respondents were happy to use it again:

The most important thing is that it is able to protect us from getting infected…

Junior doctor

With regards to device functionality, all respondents utilized bidirectional audio-visual communication, 23 (82.1%) used EHR data MR composites, 21 (75.0%) used imaging MR composites, and 16 (57.1%) used interactive MR tools such as 3D object annotation. Overall, 21 (75.0%) users agreed the device was easy to set up and 20 (71.4%) thought it was comfortable to wear:

To be honest I forget I am wearing it most of the time.

General medicine consultant

In total, 22 (78.6%) staff agreed that the HoloLens2 led to a quicker round, 21 (75.0%) a more efficient round; 22 (78.6%) had a better experience of undertaking care when using the device and 19 (67.9%) felt more engaged with the round and clinical decision making. The majority agreed that the device improved the quality of patient care (n=19, 67.9%) and that it enabled staff to make better clinical decisions (n=17, 60.7%). Only a single respondent (3.6%) reported that it reduced the quality of care, and 2 (7.2%) participants thought it did not help support better decision making. Most agreed that the HoloLens2 improved the quality of communication within the clinical teams (n=20, 71.4%), teamwork (n=23, 82.1%), and clinical situational awareness for staff while reviewing patients (n=19, 67.9%):

You can really see a clear picture so we can fully see all the signs, which on a crowded ward round you may not when you are stuck at the back somewhere.

Junior doctor

Overall, 21 (75.0%) respondents could see the clear benefit in using the technology, and 15 (53.6%) respondents agreed that it should be used for all ward rounds.

## Discussion

### Principal Findings

This technology-led pilot project has demonstrated that wearable MR devices may have a role to play in protecting staff and reducing PPE use during a pandemic response. The use of the HoloLens2 led to an 83.1% reduction in PPE use and a 51.5% reduction in the time spent by clinical staff in high-risk areas. Nearly 90% of staff felt that their clinical team was safer when using the HoloLens2 to care for COVID-19–positive patients. This feedback suggests that these material improvements in safety and PPE use do not impact the quality nor consistency of care provided and may even enhance aspects of multiprofessional care that have been hampered due to restrictions and changes in practice resulting from COVID-19. The provision of first-person, hands-free audio and visual communication across a distributed team, together with the ability to introduce relevant health data via MR composites is an important development in telemedicine and has the potential for far wider applicability outside of the immediate response to COVID-19.

### Limitations

This project suggests that substantial benefits can be obtained through the wider roll-out of MR-based technology; however, this pilot project is not without its limitations. The technology has been deployed in a digitally mature organization and across clinical areas led by motivated and interested staff. As such, there is a risk of early adopter bias, a deployment timeline that is not universally achievable, and the potential for this enthusiasm to have influenced the reported outcomes and provide an overestimation of its usability and practicality. In addition to this, the deployment within a single organization in a nonblinded, randomized, or controlled fashion may have led to the introduction of further bias and implications for the wider applicability of the findings. Further to the limitations in the methodology of this pragmatic pilot study, some issues with the technology were also highlighted. These included limited battery life, concerns around remote participants adequately hearing those not wearing the device (eg, patients), and stability and connection problems related to network speed and capacity. A key concern articulated by some users was that by providing distributed care, team members do not spend direct time with patients, which in turn may have negative consequences for future interactions and the overall quality of the doctor-patient relationship. Despite these valid concerns, patients themselves recognized the benefits presented by the technology:

They are not only saving me… I am not passing anything onto them or their friends.

Patient

In order to realize the potential benefits of this technology fully, it is vital that further work is undertaken to better understand all aspects of its impact on care delivery, staff safety, and patient outcomes. COVID-19 has led to a rapid expansion in the use of telemedicine and other digital technologies, but much of this has been understandably done at pace, without robust evaluation or assessment strategies, and often with little evidence for its safety, efficacy, and cost-effectiveness [21,22]. There is a need to demand a standardized process for effective governance, as well as robust and transparent evaluation strategies that encompass all aspects of the technology and how it impacts patients, hospitals, and the staff that use it. In addition, there is a need to develop what is an off-the-shelf solution further so that it can better meet the context-specific aspects of in-hospital care; for example, the production of device-specific protective face shields or the development of clinically focused software that allows more user-friendly blending of health data MR composites. The current ad-hoc use of generic software, while meeting the overall objective, limits future applications and potential impact.

### Comparison With Prior Work

The use of electronic or smart PPE has been identified as having the potential to protect staff and conserve resources, while simultaneously providing rapid access to emergency care [11]. However, these have largely focused on mobile or desktop solutions rather than devices which are truly wearable, and often involve ad-hoc technology fixes. The use of hands-free MR technology allows for objective improvements in communication and situational awareness for all members of the team. Enabling remote MR-supported clinical assessments can enhance the ability of team members to recognize and respond to changes in a patient’s condition over and above that offered by more basic voice or video technologies [23]. The provision of first-person, real-time audio and visual information allowed team members to “get a feel” for the patient they are consulting on despite physical distance. This was particularly evident for more junior members of the team, who may previously have not been directly at the bedside during consultations. Further to providing equivalent, if not enhanced, quality of care, obviating the physical presence of the majority of the clinicians in the ward significantly reduced the use of PPE. This is key to rationalize PPE use and may mitigate the significant supply chain disruption and equipment shortages that have been seen globally during the pandemic [24]. In addition to reducing the time clinicians spent exposed to harm and the amount of PPE used, the devices further minimized the risk of disease transmission by removing the need to handle equipment physically, such as computers, in the clinical environment. Even with proper hand sanitization and PPE use, there is still the potential for contamination of portable medical equipment [25], and so reducing physical interaction is key in minimizing risk. Finally, it is estimated that up to 7% of health care workers may have an asymptomatic COVID-19 infection [26], and around 20% of COVID-19 infections in hospital inpatients are thought to be nosocomial [27]; the use of technology to reduce exposure may therefore also act to protect patients as well as staff from disease spread.

When embarking on rapid response projects such as this, it is important to select a relatively mature off-the-shelf technology and only seek to make minor modifications. This ensures that there is background technical knowledge and awareness to help speed up deployment and aid with troubleshooting. In addition, by choosing or modifying existing technologies, issues pertaining to data security and privacy should have already been explored and understood to some extent. Agility is needed in complying with data governance regulations when they themselves may rapidly change in response to a crisis [20]. However, once the initial response is complete and the transition to a mature embedded technology platform begins, an in-depth governance and security assessment must be undertaken to ensure full compliance with regulation and legislation, and to maintain the trust of users and patients. Choosing a mature technology also means that there may be some existing evidence for its efficacy and safety, although this is often not the case with new digital technologies [28]. It is essential to ensure that deployment is context specific as no two clinical areas possess the same workflows, structures, and team practices. Final strategies for deployment and alignment to current workflows need to be flexible and driven by the clinical end-users. A clear evaluation plan must also be developed to ensure that efficacy, safety, and impact are captured and disseminated rapidly and robustly. This will ensure that time and resource are not squandered on failing technologies, and potential benefits and successes are rapidly spread both locally and more widely. Linked to this requirement for robust evidence is a need to examine patient-reported outcomes and measures of impact. It is crucial to ensure that patients perceive the quality and experience of their care to be at least maintained, if not improved with the deployment of new technologies, which is an area that was not explored in detail in this pilot study.

Contamination rates of personal devices in hospital can be over 30% and contribute to the risk of pathogen transmission [29]. The HoloLens2, like any head worn technology, is reusable, and the risk of contamination is high. Therefore, when deployed in high-risk areas, particularly when airborne disease transmission is of greatest concern, effective protocols for cleaning and decontamination need to be developed and implemented. Clear and easy-to-follow protocols for donning and doffing of the equipment must be developed locally; effective training should be delivered to ensure that staff minimize the risk of device contamination and do not put themselves at risk of self-contamination and potential infection when using off the device [30,31]. It is important to include all end-users and subject matter experts in infection control when developing and implementing these procedures to ensure they are evidence-based and that they pragmatically balance risk against ease of implementation and use to ensure optimal adherence and impact [32].

### Conclusions

This pilot nested cohort study has shown that new technologies such as the HoloLens2 have a potentially important role to play when delivering care to patients with COVID-19, minimizing staff and patient exposure to nosocomial infection risk, optimizing the use of PPE, and enhancing aspects of care. This technology has empowered a diverse group of clinicians to collaborate more effectively and efficiently, improved the transfer and dissemination of information and knowledge, and allowed care to be delivered more safely with reduced PPE demands. The initial experience of using the HoloLens2 in high-risk clinical areas is promising. What is now required is further development to cement the technology in day-to-day practice and the evolution of bespoke tools and applications that will enhance its capabilities. These developments must be coupled with robust objective evidence for its safety and effectiveness across a range of settings to ensure its impact on staff, patients, and hospitals is fully realized.

## Appendix

Multimedia Appendix 1 Suggested protocol for donning/doffing and decontamination of the HoloLens2 device.

## Abbreviations

COVID-19: coronavirus disease
EHR: electronic health record
MAC: media access control
MR: mixed reality
NHS: National Health Service
NIHR: National Institute for Health Research
PPE: personal protective equipment
QI: quality improvement
