# Supplementary material for: Use of the HoloLens2 Mixed Reality Headset for Protecting Health Care Workers During the COVID-19 Pandemic: Prospective, Observational Evaluation
Source: J Med Internet Res. 2020 Aug 14;22(8):e21486. doi: 10.2196/21486 (PMC7431236; doi:10.2196/21486)
Supplement: Multimedia Appendix 1 [file jmir_v22i8e21486_app1.pdf]

HOLOLENS - Infection Prevention and Control

Summary

The use of HoloLens™ has been reviewed and approved by the Infection Control Technical Advisor and IPCC Team. A pragmatic, risk-benefit based process for using and decontaminating the device has been agreed. This process is aligned with that used for the decontamination of reusable items of PPE such as full-face plastic shields.

- The user will wear full PPE as per local guidance
- A surgical cap/net is to be worn and the device placed over the top. A second surgical cap/net is then to be placed over the device so as to provide full coverage but not obscure the front facing cameras - as shown below
- The device provides some eye protection that is sufficient for conducting routine clinical work in COVID+ve areas such as ward rounds or other non-aerosol generating procedures; individual users should make a dynamic risk assessment at the time of use. It is possible to modify a full-face shield for use with the device
- The device is appropriate to wear for sessional work as per local PPE guidelines
- Once the clinical session is complete the device can be removed in-turn as part of the standard doffing procedure
- The device should be immediately decontaminated after use with an appropriate disinfecting wipe (e.g. Clinell® Universal Wipe)

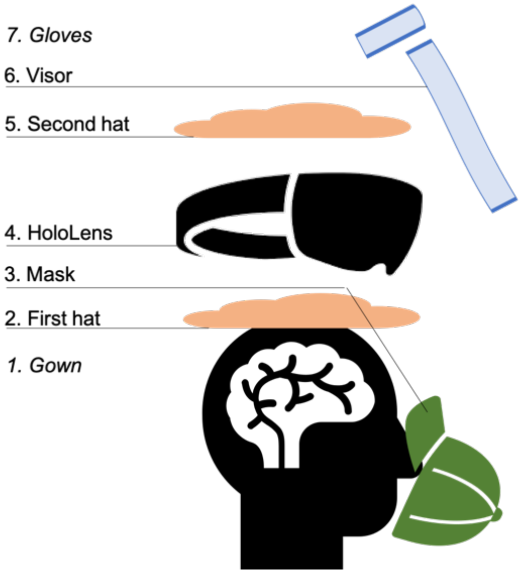

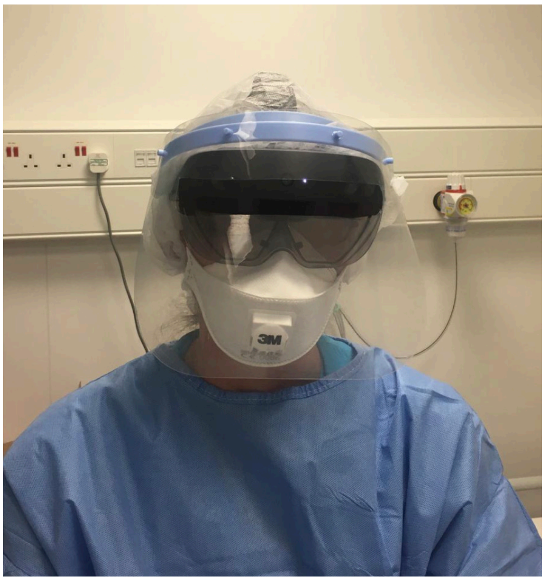

Donning Process

The device is appropriate to wear sessionally - mask/respirator, visor, gown and HoloLens™ can remain in-situ, but apron and gloves should be changed between every patient.

1. Apply apron/long sleeve fluid repellent gown and fluid repellent facemask/respirator as per local SOP
2. Put on a single use surgical cap and place the device over the top. Adjust the device to ensure a comfortable fit and the visor is aligned correctly
3. Place a second single use surgical cap over the top of the device so as to provide full coverage but not to obscure the front facing cameras/sensors
4. A specific full-face visor can then be put on if required
5. Apply second set of gloves

Doffing Process

1. Remove outer pair of gloves. Sanitise hands
2. Remove full face visor (if worn) and outer surgical cap. Sanitise hands
3. Remove HoloLens™ device. Sanitise hands
4. Remove inner surgical cap, apron/gown and inner gloves. Sanitise hands
5. Remove mask/respirator
6. Wash hands

Decontamination Process

1. Ensure wearing appropriate PPE (apron, gloves, eye protection)
2. Check device for damage
3. Clean device in an S-shape motion with disinfectant wipe
    a. 1st wipe - inside of device starting with lens area nearest the eyes
    b. 2nd wipe - outside of device starting with the lens area nearest the eyes
4. Leave device to dry on clean decontaminate surface. Once dry store in case
5. Remove PPE and wash hands
